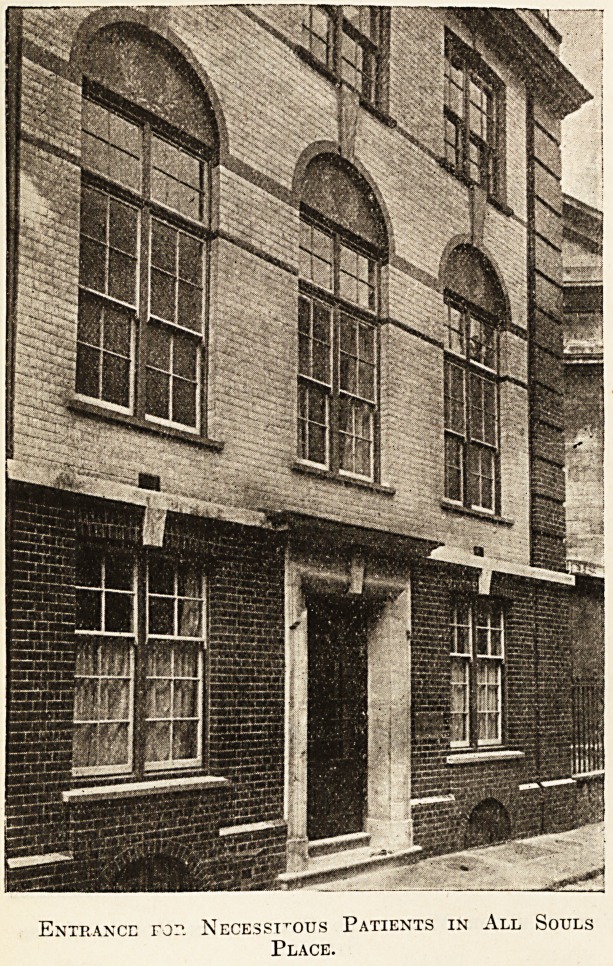# The New Radium Institute

**Published:** 1911-08-19

**Authors:** 


### THE NEW RADIUM INSTITUTE.

### ITS ORGANISATION, APPLIANCES, AND STAFF.

Before hearing one word of description concerning the Radium Institute, which was opened on Monday last in Riding House Street, Portland Place, it is very important that hospitals and the general public should realise, beyond the possibility of further misapprehension, that patients can be treated only in association with, and through the introduction of, their medical adviser. The medical superintendent, Mr. A. E. Hayward Pinch, F.R.C.S., on whom for the past two years practically all the responsibility of organising the new Institute has been cast, has been deluged for the past few weeks with a mass of correspondence, which would never have been despatched had the public grasped this simple and irrefragable rule. The greatest part of the net profit on the postage of letters which His Majesty's Postmaster-General records each year is due probably to a daily repetition of similar public misunderstandings, but that is a poor consolation to the victims, who, like Mr. Pinch, have more important matters on hand than the elementary education of the public.

As everyone now knows, the Radium Institute in London has been founded through the generosity of Lord Iveagh and Sir Ernest Cassel. It enjoys an invested income, which will be supplemented by the fees of those well-to-do patients who can afford the following scale: For the first consultation, £2 2s., the patient being accompanied by his or her medical adviser, or bringing from such medical adviser a written history of the case; for treatment (*a*) when the lesion is situated on the external surface of the body either £2 2s. or £4 4s., according as the apparatus contains more or less than 50 milligrammes of radium per sitting; (*b*) when the lesion is situated in a cavity, £5 5s. per sitting. All fees must be paid at the time of attendance, and the printed regulations state that "medical practitioners are cordially invited to be present during the treatment of their patients, and to assist in the same whenever practicable."

On the other hand the endowment of the Institute allows it to extend its treatment to those who cannot afford to pay, provided always that their application be made through a medical man, and that their "necessity" is vouched for on his signature. Only so can cases unsuitable alike ex natura and ex accidenti be excluded. The Institute accordingly has one function to perform for two classes, the treatment of cases suitable for radium therapy in both rich and poor. This function has been recognised by the architect, Mr. T. P. Figgis, F.R.I.B.A., and he has designed the building for the reception of the two classes of sufferers.

### The Building Described.

The accompanying photographs give a good idea of its external appearance. The first has been taken from Riding House Street, and shows the entrance by which the well-to-do patients will enter. Immediately behind this entrance door and on the opposite, or rear, side of the building is All Souls Place, by an entrance door in which the necessitous patients will be admitted. The ground floor consequently is bisected by a door which divides the two classes of patients from each other. The only difference likely to strike the observer between the rooms allocated to the well-to-do and to their poorer fellows is that the furniture in the rooms of the former is of mahogany and in the latter of

Entrance for Well-to-do Patients in Riding House Street.

oak. In the degree of comfort afforded, both classes are equally well looked after.

The ground floor, then, consists of two waiting-rooms, two inquiry offices, the office of Mr. Pinch, the medical superintendent, four consulting rooms, and a gynæcology room. Below this floor, to reverse the usual order, is the basement, which provides space for two strong rooms, a carpenter's shop, the boiler room, with its five water systems, each with a function of its own, and lastly a balance room. This last is interesting because it contains the necessary balance of the Bungë pattern, the exceptional delicacy of which is secured by its position on twin piers which are built directly upon the foundations. The motor omnibus above and the tube below are alike impotent to affect it by their vibrations.

The ground floor, which we have described already, may be summed up shortly as the floor devoted to diagnosis. Communication between the basement and the floors above it is made by means of a staircase and an automatic lift—an "Otis elevator," as the Americans would say. Mounting by one or the other the first floor is reached, and is found to consist of twelve cubicles for treatment, comprising four for "dark" cases. All the cubicles are simple and comfortable, if simply comfortable is not a better way of putting it.

The first floor, in short, is given up to treatment.

Entrance for Necessitous Patients in All Souls Place.

The second, which is designed for research, contains, at the All Souls' end, a chemical laboratory, three photography rooms, one dry and one wet dark room, and a smaller pathological laboratory. At the Riding House Street end are the Board Room, Stenographers' Room, and Common Room for the staff.

On the third floor is the mechanical workshop, which is presided over by Mr. John F. Holding, the "technical assistant," who was trained under Sir William Ramsay, and whose pleasant boast it is that his shop can turn out practically anything likely to be required of it; the sitting-room for the charge nurse, Sister Olsen, who, if not, as she probably is, the only nurse in this country who has had an expert training in the nursing of radium-treated cases, is certainly the only one trained under the eye of the great Finsen himself; and finally the medical superintendent's private study, to which he is able to escape for a moment from the present overwhelming correspondence to which we have alluded above. The fourth floor alone remains, and as that contains the private rooms of Mr. Pinch, of it nothing shall be said.

### The Appliances.

So much for the general plan of the building. As, however, it is supposed, and no doubt correctly supposed, that the new Institute contains the latest discoveries of applied science, the departments above described demand some further detail. Returning to the basement, therefore, we find that the construction of the strong-room presented certain problems idiosyncratic to the substance which was to be stored, and the solution of these is both popularly impressive and, in at least one respect, ingenious. Its walls are made of eighteen inches of blue Dorset brick; its floor is of concrete, flush or piled up on the foundations. Eight inches of concrete have gone to the making of its roof. The room looks like a larder, with the slate or lead shelf that we associate with the storage of less penetrating radio-active substances than radium, and in order that those concerned to check the return, or amount, of the precious supply within, may not endanger their epidermis by unavoidable contiguity on entering, a special system of pigeon-holes is being devised by Mr. Holding, the peculiar value of which will lie in the automatic mechanism which he is designing to indicate from the strong-room door the presence or absence of the radium.

The simple diagnostic aim of the ground floor requires no further elaboration; nor does the therapeutic arrangement of cubicles on the one above it. It is only when we come to the research laboratories on the second floor that detail has any instructive value.

Taking first the pathological laboratory, one notes, among the classic apparatus, a Minot's microtome for section-cutting to the 1-25,000 of an inch; the inevitable Hearson's incubator, and Chamberlen's autoclave for the sterilisation of the media in which the bacteria will be cultivated. There is, too, an embedding bath with Reichert's mercury thermo-regulator, a Zeiss Nernst lamp with luminous zirconia filament, and a Zeiss microscope with apochromatic lenses. In the photographic room on the same floor is a camera with a Zeiss-Tessar F 4.5 lens, and in addition a Kodak "Aristo" grand studio camera and a Kodak studio arc lamp of about 10,000 candle-power. The most notable feature of the accompanying dark room is the relatively new electrical printer which Kodaks have supplied, and which allows a plate to be printed, exposed, or subjected to various coloured rays at pleasure. The dark-room also satisfies Mr. Holding's private test that the red lamp should be powerful enough to allow a newspaper to be read with comfort, and, incidentally, to be of practical value to the photographer by allowing him to see what he is about.

The well-lighted, relatively large chemical laboratory is very much like any other, with as many beakers, retorts, and test-tubes as Rossetti managed to crowd so delightfully into his picture with that title. There is, however, an Antropoff mercury pump, a Russian invention for producing high vacua, which was, presumably, unknown to Rossetti's mediæval chemist and astrologer.

Of the mechanical workshop on the third floor not very much need be said. Beyond the usual mechanical tools and various instruments of precision, screw-cutting appliances, and what not, we may yet note the inevitable Drummond lathe, which will perform a variety of tasks, a Parkinson vice, and a set of carborundum grinding wheels of excruciating hardness. This must conclude our brief survey of the mechanical furniture of the Institute.

### The Staff.

Of the staff this much should be said: The donors of the Institute, Lord Iveagh and Sir Ernest Cassel, are its two presidents. There is also an executive committee, of which Sir Frederick Treves is chairman. This committee meets at intervals, and consists of the Hon. Walter E. Guinness, M.P., Felix Cassel, Esq., K.C., M.P., the Hon. Robert J. Strutt, F.R.S., Sir Lauder Brunton, Bart., F.R.S., Sir William Ramsay, K.C.B., F.R.S., Sir Malcolm Morris, K.C.V.O., and Sir Joseph John Thomson, F.R.S. To this committee Mr. Pinch, as medical superintendent, is responsible, though we understand that it has allowed him a free hand, being not forgetful of the organising and technical ability which he displayed in the old days at the Polyclinic, Chenies Street. The director of the chemico-physical laboratories is Mr. W. Lester Alton, F.I.C., whose technical assistant, as stated, is Mr. J. F. Holding. So much for the heads of the executive staff. In addition there is a nursing staff of at present one male and two female nurses, with Sister Olsen as charge nurse.

As the work is of course entirely out-patient, there is no sleeping accommodation for the staffs, nor are the members able to dine on the premises. Rumour says that it has been laughingly suggested that the oak-panelled board-room on the second floor invites adaptation to this or some less reverend purpose than its present magnificence would be content with. But of that we cannot say.

It remains only to add that well-to-do patients are received on Tuesdays and Thursdays; the necessitous on Mondays, Wednesdays, and Fridays. Treatment, however, goes on all the week round, and, finally, patients, with the essential proviso stated in the opening words of this account, are received only by appointment.

## Figures and Tables

**Figure f1:**
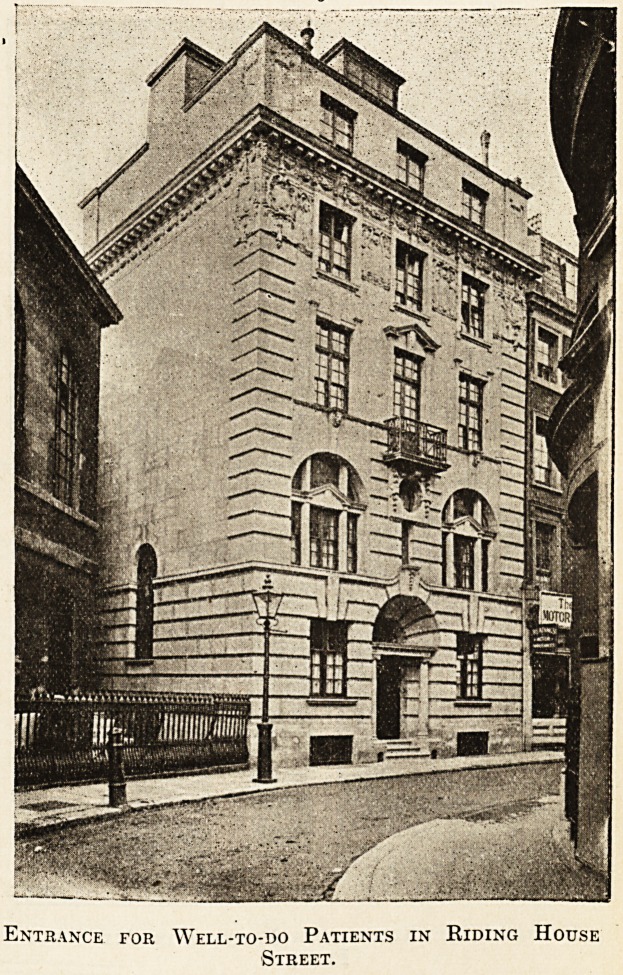


**Figure f2:**